# Transmural migration of a surgical compress into the stomach after splenectomy: a case report

**DOI:** 10.4076/1757-1626-2-7975

**Published:** 2009-07-30

**Authors:** Sami Akbulut, Mert Mahsuni Sevinc, Fatih Basak, Sefika Aksoy, Bahri Cakabay

**Affiliations:** 1 Department of Surgery, Diyarbakir Education and Research HospitalOp. Dr. Seref Inaloz Caddesi 21400, DiyarbakirTurkey; 2 Department of Surgery, Istanbul Education and Research HospitalK.M. Pasa, 34321, IstanbulTurkey

## Abstract

A surgical compress retained in the abdominal cavity following surgery is a serious problem. Here, we describe a 33-year-old female who was admitted with abdominal pain, vomiting, no passage of gas or feces, and abdominal distension for 3 days. She had a splenectomy at another medical center 4 years previously. An upright plain abdominal film revealed small bowel obstruction with marked small bowel air-fluid levels. The physical examination revealed muscular guarding and rebound tenderness in the periumbilical region. Therefore, a laparotomy was performed. A surgical compress was removed at enterotomy and the final diagnosis was gossypiboma. Because a retained surgical compress may lead to medicolegal problems, it is important to count the material used before and after a surgical procedure to reduce the risk of this problem.

## Introduction

Gossypiboma is the term used to describe a surgical swab retained after surgery. Synonyms are textiloma and cottonoid [1]. Fortunately, cases where instruments or sponges are left behind following a surgical procedure are uncommon, although they are potentially dangerous medical errors [2,3]. The literature estimates that a foreign body is retained after intra-abdominal surgery in 1:1,000 to 1:1,500 cases [2,3]. Here, we present a case of transgastric gossypiboma after a splenectomy 4 years previously.

## Case presentation

**Figure 1.**
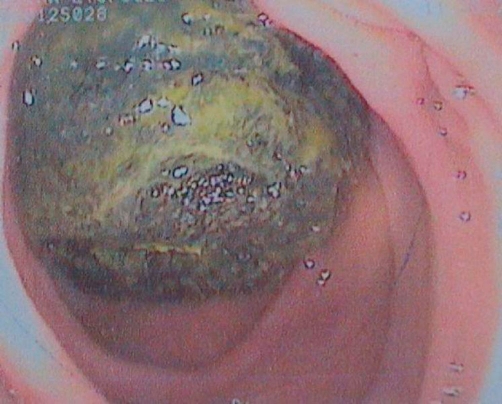
A mass compatible with a phytobezoar was observed in the duodenum via endoscopy.

**Figure 2.**
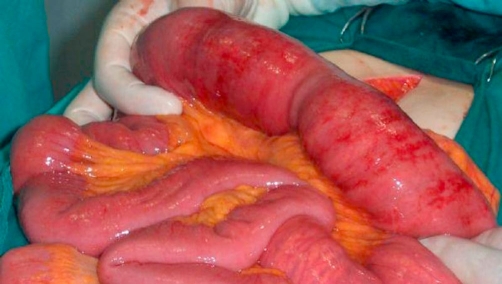
A mass obstructing the lumen of the ileum was observed during laparotomy.

**Figure 3a, b and c.**
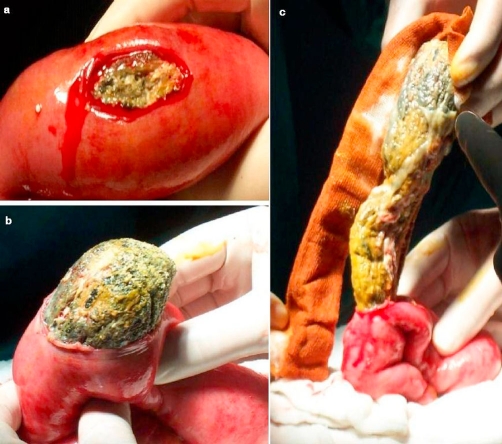
The compress was removed at enterotomy.

A 33-year-old Turkish woman was admitted to our emergency unit with abdominal pain, severe nausea and vomiting, constipation, no passage of gas or feces, and abdominal distension for 3 days. She had a splenectomy at another center 4 years previously. The previous year, endoscopy had been performed because of dyspepsia and stomach ache. A peptic ulcer crater and a mass compatible with a phytobezoar in the duodenum were observed via endoscopy (Figure 1). Laboratory investigations showed blood urea nitrogen at 38 mg/dl, creatinine at 1.2 mg/dl, and C-reactive protein at 45 mg/l. The blood cell count revealed leukocytosis at 17.500/μl, with a hemoglobin level of 12.9 g/dl, and a platelet count of 327,000/μl. Other serum parameters were within normal limits. The physical examination revealed muscular guarding and rebound tenderness in the periumbilical region. An upright plain abdominal film revealed small bowel obstruction with marked small bowel air-fluid levels. No pathological findings were observed via ultrasound because of distension. The patient underwent an emergency laparotomy with a midline incision. At laparotomy, a mass was observed obstructing the intestine completely 150 cm distal to the ligament of Treitz (Figure 2). A 15 × 20 cm surgical compress was removed at enterotomy (Figure 3a, 3b, 3c). The patient had an uneventful postoperative course and was discharged on the fifth postoperative day.

## Discussion

Postoperative surgical complications are frequently unavoidable. However, some complications result from human error in both the intra- and postoperative periods. One such complication, which is frequently under-reported, is a retained compress or gossypiboma [4]. Retained surgical sponges can cause serious consequences, such as bowel or visceral perforation, obstruction or fistula formation, sepsis, or even death [2,5]. Intra-abdominal gossypibomas can migrate into the ileum, stomach, colon, or bladder without any apparent opening in the wall of these luminal organs [6]. Once in the gastrointestinal system, they cannot pass through the ileocecal valve and cause complete or incomplete intestinal obstruction at this level [7]. In this case, a surgical compress was observed in an ulcerative lesion where it had passed through the stomach wall. It took the compress about one year to pass from the stomach and cause obstruction in the ileum. No abscess or fistula in the abdomen was found.

Gossypiboma is difficult to diagnose, but plain X-rays, ultrasonography (US), computed tomography (CT), and magnetic resonance imaging (MRI) can help to make the diagnosis [8]. In this case, air-water levels were observed via plain X-ray, whereas no pathological finding was observed via US. Typically, gossypibomas are discovered in the first few postoperative days, although they may remain undetected for many years. Bowel obstruction, perforation, pseudotumor, and peritonitis are the most common clinical presentations, although in some cases constitutional symptoms prevail [9]. Tumerve *et al*. [10] examined 10 cases: eight involved sponges and two involved clamps. In their series, the patients first became symptomatic between 15 days and 20 months postoperatively. Five of their patients had previous Caesarean sections or hysterectomies. Yildirim *et al*. [11] investigated 14 patients who were between 25 and 79 years of age. In their group, the initial complaints began between 5 days and 40 years postoperatively (the last case was an open cholecystectomy). Most of the patients were admitted to hospital with abdominal pain or intestinal obstruction. In our case, the obstruction developed 4 years postoperatively.

In conclusion, small sponges should not be used during laparotomy. Compresses should only be used intraperitoneally, one at a time, mounted on forceps. Before closing the peritoneum, the surgeon should explore the abdominal cavity completely.

## Abbreviations

CT: Computed tomography
MRI: Magnetic resonance imaging
US: Ultrasonography
